# Analyses of Insecticide Resistance Genes in *Aedes aegypti* and *Aedes albopictus* Mosquito Populations from Cameroon

**DOI:** 10.3390/genes12060828

**Published:** 2021-05-28

**Authors:** Borel Djiappi-Tchamen, Mariette Stella Nana-Ndjangwo, Konstantinos Mavridis, Abdou Talipouo, Elysée Nchoutpouen, Idene Makoudjou, Roland Bamou, Audrey Marie Paul Mayi, Parfait Awono-Ambene, Timoléon Tchuinkam, John Vontas, Christophe Antonio-Nkondjio

**Affiliations:** 1 Vector Borne Diseases Laboratory of the Applied Biology and Ecology Research Unit (VBID-URBEA), Department of Animal Biology, Faculty of Science, University of Dschang, P.O. Box 067 Dschang, Cameroon; bamou2011@gmail.com (R.B.); mayimariepaulaudrey@yahoo.com (A.M.P.M.); timotchuinkam@yahoo.fr (T.T.); 2 Institut de Recherche de Yaoundé (IRY), Organisation de Coordination pour la lutte Contre les Endémies en Afrique Centrale (OCEAC), P.O. Box 288 Yaoundé, Cameroon; stellanana123@gmail.com (M.S.N.-N.); atalipouo@gmail.com (A.T.); enchoutpouen2002@yahoo.fr (E.N.); idenemakoudjou@mail.com (I.M.); hpaawono@yahoo.fr (P.A.-A.); 3 Department of Animal Physiology and Biology, Faculty of Science, University of Yaoundé I, P.O. Box 337 Yaoundé, Cameroon; 4 Institute of Molecular Biology and Biotechnology, Foundation for Research and Technology-Hellas, 70013 Heraklion, Greece; mavridiskos@gmail.com (K.M.); vontas@imbb.forth.gr (J.V.); 5 Pesticide Science Laboratory, Department of Crop Science, Agricultural University of Athens, 11855 Athens, Greece; 6 Department of Vector Biology, Liverpool School of Tropical medicine, Pembroke Place, Liverpool L3 5QA, UK

**Keywords:** *Aedes aegypti*, *Aedes albopictus*, insecticide resistance diagnostics, arbovirus, mechanisms, urban settings, Cameroon

## Abstract

The emergence of insecticide resistance in *Aedes mosquitoes* could pose major challenges for arboviral-borne disease control. In this paper, insecticide susceptibility level and resistance mechanisms were assessed in *Aedes aegypti* (Linnaeus, 1762) and *Aedes albopictus* (Skuse, 1894) from urban settings of Cameroon. The F1 progeny of *Aedes aegypti* and *Aedes albopictus* collected in Douala, Yaoundé and Dschang from August to December 2020 was tested using WHO tube assays with four insecticides: deltamethrin 0.05%, permethrin 0.75%, DDT 4% and bendiocarb 0.1%. TaqMan, qPCR and RT-qPCR assays were used to detect kdr mutations and the expression profiles of eight detoxification genes. *Aedes aegypti* mosquitoes from Douala were found to be resistant to DDT, permethrin and deltamethrin. Three kdr mutations, F1534C, V1016G and V1016I were detected in *Aedes aegypti* populations from Douala and Dschang. The kdr allele F1534C was predominant (90%) in *Aedes aegypti* and was detected for the first time in *Aedes albopictus* (2.08%). P450s genes, Cyp9J28 (2.23–7.03 folds), Cyp9M6 (1.49–2.59 folds), Cyp9J32 (1.29–3.75 folds) and GSTD4 (1.34–55.3 folds) were found overexpressed in the Douala and Yaoundé *Aedes aegypti* populations. The emergence of insecticide resistance in *Aedes aegypti* and *Aedes albopictus* calls for alternative strategies towards the control and prevention of arboviral vector-borne diseases in Cameroon.

## 1. Introduction

Mosquitoes of the *Aedes* genus particularly *Aedes aegypti* and *Aedes albopictus* are major vectors of five important arboviral diseases worldwide (dengue, chikungunya, rift valley fever, yellow fever virus and zika) [1,2]. *Aedes aegypti* originates from Africa, whereas *Aedes albopictus* originates from South East Asia [3,4]. These two species, which now overlap in most of their distribution range appear to be well adapted to the urban environment [5,6]. In recent decades, arboviral diseases such as dengue and chikungunya have been increasingly reported across sub-Saharan Africa with important outbreaks reported in major urban settings [7,8,9,10,11,12,13,14,15]. These changes in the epidemiology of arboviral diseases could be closely linked to the co-occurrence of these two competent vector species in most epidemiological settings [16,17,18,19,20]. In Cameroon, frequent occurrence of arboviruses outbreaks or sporadic cases of yellow fever, dengue, chikungunya and Zika were reported in the early 2000s, a few years after the introduction of *Aedes albopictus* in the country [21,22,23,24,25,26,27]. Due to the lack of effective drugs and vaccines against most of these arbovirus diseases, vector management is the main strategy for reducing transmission and preventing outbreaks [28,29]. Approaches for controlling *Aedes* spp vector populations include active community participation in vector control interventions, health education programs, reduction of breeding sites, environmental management, improvements of water supplies and storage, solid waste management, modification of human-made larval habitats and insecticide use [30,31,32,33,34]. Insecticide-based intervention is the main strategy routinely used to control mosquito populations [35]. Despite the increasing nuisance due to *Aedes* mosquitoes bites, there have been so far limited control efforts targeting specifically *Aedes* mosquitoes populations. Most vector control efforts are directed against malaria vectors with the massive deployment of insecticide-treated nets [36]. It is likely that the scaling up of insecticide-treated nets alongside the intensive use of insecticides in agriculture could be affecting non-malaria vector species [37] and could lead to the development of resistance within *Aedes* mosquito populations [20,38,39]. Mosquitoes may display one or more resistance mechanisms, making them less susceptible to insecticides [40]. These include behavioral changes [41,42], alterations of the cuticle to reduce insecticide penetration [43,44,45,46], target site resistance [41,42] and increased detoxification metabolism involving genes such as cytochrome P450 monooxygenases (P450s), carboxylesterases (COEs) and glutathione S-transferases (GSTs) [47,48,49,50,51,52].

Several cytochrome P450s (CYPs), more often members of the CYP6 and CYP9 families, have been associated with resistance in *Aedes* vectors [53,54,55]. The *AaegCYP9J28* and the *AaegCYP6BB2* are detected more often and consistently across studies in *Ae. (Aedes) aegypti. AaegCYP9J32* has been associated with pyreroid resistance in Thailand, Mexico and Vietnam; *AaegCYP9J24* and *AaegCYP9J26* in Latin America and Singapore; and *AaegCYP9M6* and *AaegCYP4D24* in Asia and Puerto Rico, respectively [55]. The *AalCYP6P12* has been associated with pyrethroid resistance in *Ae. albopictus* populations from Malaysia [56].

Mutations in the voltage-gated sodium channel (VGSC) are common in *Ae. aegypti,* with 10 mutations at eight codon positions in VGSC domains II–IV identified to date [53]. Their geographical distribution and frequency vary: the most widespread mutation in both *Ae. aegypti aegypti* and *Ae. aegypti formosus* is the 1534C across continents. The V1016I and V1016G mutations have been also found in Asia, in the Americas and in Africa [53].

Previous studies examining general resistance status of the two main vectors *Ae. aegypti* and *Ae. albopictus* in different ecological settings across Cameroon indicated that *Aedes albopictus* and *Aedes aegypti* were both resistant to 0.05% deltamethrin, 0.01% bendiocarb and 4% DDT (dichlorodiphenyltrichloroethane). Furthermore, they were recorded to be partly susceptible to 0.75% permethrin and fully susceptible to malathion 5% [38,39,57,58]. Although pre-exposure of mosquitoes to the synergist PBO (piperonil butoxide) or DEM (diethyl maleate) increased mosquito susceptibility status to permethrin, deltamethrin and DDT, it is not clear which detoxification genes are involved in insecticide metabolism as well as additional mechanisms involved in *Aedes* resistance to insecticides.

In the present study, the insecticide resistance profile of adult *Ae. aegypti* and *Ae. albopictus* from three different urban settings was determined by WHO bioassays, and subsequently, the underlying resistance mechanisms were investigated using molecular tools, to detect genes and mutations associated with insecticide resistance in these vector populations.

We tested the hypothesis that *Aedes* populations across Cameroon could display a similar resistance profile (% mortality) and similar resistance gene frequencies.

## 2. Materials and Methods

### 2.1. Study Sites

**Figure 1:**
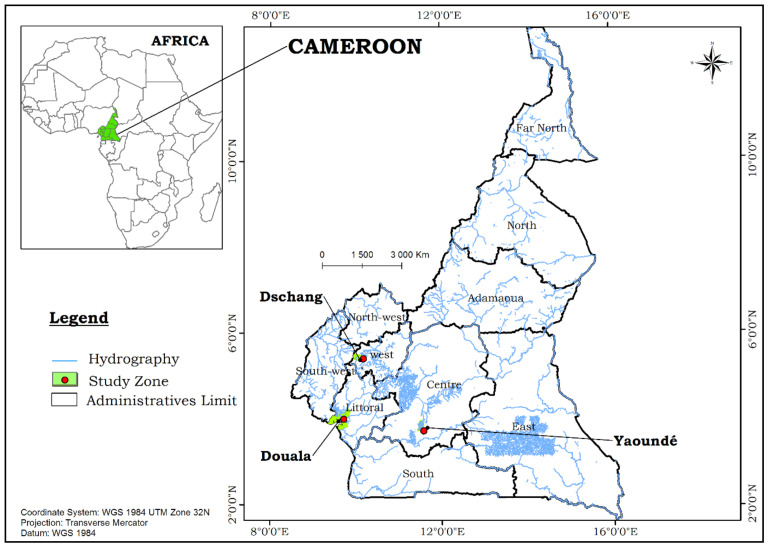
Localization of the study sites in Cameroon.

The study was carried out in three cities of Cameroon (Figure 1), namely: Yaoundé (equatorial forest region), Douala (coastal region) and Dschang (highland region) (Table 1). These cities are situated at different altitudes.

Mosquito sampling in Yaoundé was conducted in the districts of Mvan, Obili and Simbock. In Douala collections were done in Bonaberi, Yassa and Village while in Dschang, mosquitoes were collected in Foréké, Tsinbing and Paidground.

### 2.2. Collection of Mosquito Larvae, Rearing and Processing

Immature stages of *Aedes* mosquitoes were collected in each city from artificial breeding sites such as used tires around houses and garages, discarded plastics containers and metallic containers from August to December 2020. The collected immature stages were pooled (according to cities) and reared to adult stage under standard laboratory conditions (27–28 °C temperature; 70–80% hygrometry). Those collected in Dschang were reared at the VBID-URBEA of the University of Dschang. Samples from Douala and Yaoundé were reared in the insectary of the Malaria Research Laboratory of OCEAC. Pupae were collected daily and transferred in cages for adult emergence. Adult mosquitoes were provided continuous access to 10% glucose solution. Morphological identification of *Aedes* mosquitoes from each study site was done under a stereomicroscope with the keys of jupp (1996) [59]. A first subset of 50–60 unexposed non-blood fed mosquito females aged 3–5 days were preserved in RNA later (SIGMA Aldrich, Saint Louis, MO, USA) for characterization of molecular mechanisms of insecticide resistance. The remaining mosquito species were fed on chicken blood for egg-laying, and insecticide susceptibility bioassays were conducted with females of the F1 generation. After bioassays, survivors against all insecticides were preserved in 70% ethanol and sent to IMBB-FORTH (Greece). Mosquitoes that survived exposure to insecticides were used for kdr genotyping analysis and species identification (PCR).

### 2.3. Insecticide Susceptibility Tests

Bioassays were performed following the WHO guidelines [60] with four insecticide classes. For each mosquito population, four replicates of 20 F1 females each were exposed to insecticides impregnated papers. *Aedes* mosquitoes were exposed to 0.05% deltamethrin (only for *Aedes aegypti* population from Douala), 0.75% permethrin, 4% DDT and 0.1% bendiocarb; most of the impregnated papers are prepared by diluting the insecticide in silicone oil (solvent). *Aedes* species were exposed to these discriminating insecticide doses instead of their normal discriminating dose because they share similar habitats with *Anopheles gambiae* and *Culex quinquefasciatus* who happen to be highly resistant to insecticides, and we wanted to assess whether *Aedes* populations have similar resistance profile. Previous studies in Cameroon reported *Aedes aegypti* populations to be resistant to their normal discriminating doses [39,58]. For each bioassay, two replicates of 20 female mosquitoes unexposed to any insecticide were used as an internal control. A susceptible strain of *Ae. aegypti* and *Ae. albopictus* mosquitoes from Cameroon were used to validate the efficacy of the impregnated papers. After 60 min of exposure, mosquitoes were transferred into holding tubes (12 cm in height; 4.2 cm diameter) and supplied with 10% glucose. The mortality rate was recorded 24 h post-insecticide exposure, mosquitoes that survived exposure to insecticides were used for kdr genotyping analysis and species identification through PCR.

### 2.4. Total Nucleic Acids (NAs) Extraction from Mosquito Pools and gDNA Extraction from Individual Mosquitoes

Total NAs were extracted from pooled mosquito specimens (N = 10 mosquitoes per pool) using the MagSi magnetic beads extraction kit (Magnamedics) as previously described [61]. For gene expression analysis (RNA) mosquitoes unexposed to insecticides, non-blood-fed females, aged 3–5 days, were used. For genotyping (DNA), mosquitoes that had previously survived exposure to insecticides were used. The quantity of total NA was assessed spectrophotometrically (Nanodrop). The quality of RNA was assessed by 1.0% *w/v* agarose gel electrophoresis (Supplementary Figure S1). Genomic DNA (gDNA) from individual mosquitoes was extracted with the DNAzol (MRC, Inc., Saint Louis, MO, USA) protocol according to the manufacturer’s instructions.

### 2.5. Genotyping of Mosquito Sample and Multiplex RT-qPCR for Gene Expression Analysis

Species identification at the molecular level was performed using the TaqMan assay of Kothera et al. [62]. Previously developed and validated triplex TaqMan (RT-qPCR) assays (Supplementary Table S1) were used for the quantification of 07 detoxification genes’ expression (Cyp6BB2, Cyp9J26, GSTD4, CCEae3a, Cyp9J28, Cyp9M6 and Cyp9J32) including RPL8 for normalization purposes in each assay as previously described. Primers were designed in the exon–exon junctions for all genes, thus eliminating the need for a DNase digestion step [63]. TaqMan assays were also used for detecting kdr mutations F1534C, V1016G, V1016I and S989P in gDNA from *Ae. aegypti* and F1534C in gDNA from *Ae. albopictus* mosquitoes (Supplementary Table S2) as previously described [63]. Wild-type, mutant and heterozygous gBlocks™ Gene Fragments control sequences (IDT, Coralville, IA, USA) for each mutation (Supplementary Table S2) were included in each run to better facilitate the genotyping call [63]. Reactions were performed in the Viia7 Real-Time PCR system (Applied Biosystems, Foster City, CA, USA) using a one-step RT-PCR mastermix supplied by FTD (Fast-track diagnostics, Esch-sur-Alzette, Luxembourg) in a total reaction volume of 10 µL. The thermal cycle parameters were: 50 °C for 15 min, 95 °C for 3 min, and 40 cycles of 95 °C for 3 s and 60°C for 30 s. Samples were amplified in duplicates and each run always included a non-template control.

### 2.6. Statistical Analysis

For adult insecticide bioassays, the status of mosquitoes was defined by mortality rate: confirmed resistance if mortality <90%, possible resistance if mortality is between 90 and 98%, and susceptible if mortality >98% [60]. Calculation of fold-changes, 95% confidence intervals (CI) and statistical significance was performed according to the Pfaffl method [64]. More precisely gene expression analysis was performed using the REST© 2009 (v2.0.13) [65] software that uses a Pair-Wise Fixed Reallocation Randomization Test to statistically analyze the gene expression data. Graphs were constructed with the SigmaPlot software (v12.0).

## 3. Results

### 3.1. Insecticide Bioassays Results

**Figure 2:**
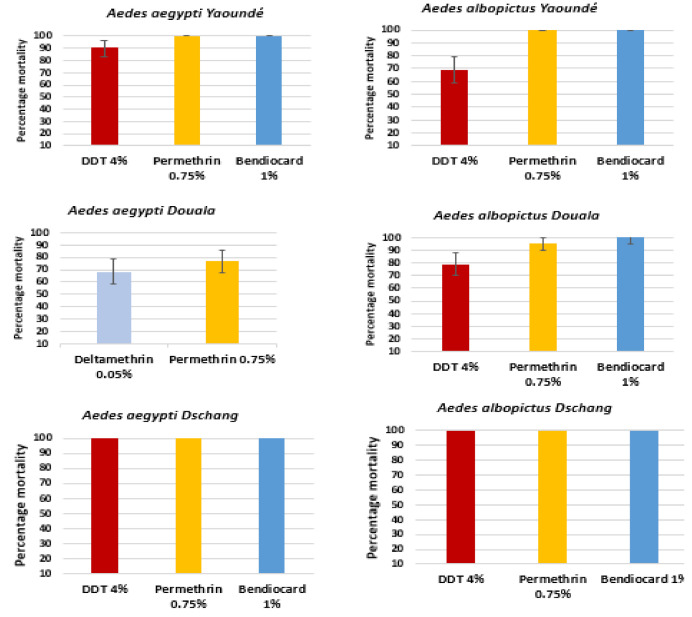
Susceptibilities profiles of *Aedes aegypti* and *Aedes albopictus* in Yaoundé, Douala and Dschang. Error bars represent 95% confidence intervals.

Adult bioassays revealed different susceptibility levels against DDT ranging from 68.75% to 100% in the three field populations of *Ae. Aegypti* and *Ae. Albopictus*. *Ae*. *Albopictus* populations were fully susceptible to permethrin and deltamethrin insecticides, while *Ae*. *aegypti* from Douala displayed high resistance to both deltamethrin 0.05% and permethrin 0.75%. Both species were fully susceptible to bendiocarb (Figure 2) in all study sites. There was no significant difference when comparing the mortality rate of *Aedes aegypti* populations between sites (*p >* 0.05). A significant difference in the mortality rate of *Aedes albopictus* populations was recorded when comparing Yaoundé to Dschang population to DDT 4% (*p <* 0.001) and for Bendiocard between Yaoundé and Douala populations (*p* = 0.03).

### 3.2. Species Identification

A total of 184 specimens were genotyped to confirm the morphological identification of *Aedes* species in each locality. A subsample of 72 *Aedes albopictus* and 72 *Aedes aegypti* identified morphologically were further processed by PCR and all turned to confirm morphological identifications.

### 3.3. Screening of Target Site Mutations (kdr F1534C, V1016G, V1016I and S989P)

The distribution of different mutations associated with insecticide resistance was assessed. In *Ae. aegypti*, a total of three-point mutations were detected (Table 2), namely F1534C, V1016G and V1016I. Among these, the mutation F1534C was highly predominant (>60%) and was detected in Douala and Dschang (Table 3). The V1016G kdr allele was detected only in the population of Douala. Furthermore, the V1016I allele was found with a frequency ranging from 26.7 to 60% in *Ae. aegypti* mosquitoes from Douala and Dschang, respectively. No mosquito was found with the S989P mutation and no mutation was found in both *Aedes aegypti* and *Aedes albopictus* in Yaoundé.

### 3.4. Analysis of Detoxification Genes Expression Profile

Quantitative RT-qPCR analyses revealed the overexpression profile of seven different detoxification genes in resistant *Aedes aegypti* from the three study sites namely, *Cyp6BB2*, *Cyp9J26*, *GSTD4*, *CCEae3a*, *Cyp9J28*, *Cyp9M6* and *Cyp9J32* (Table 4). High overexpression ratios were recorded for three P450 cytochrome genes, namely *Cyp9J28* (2.84–7.55), *Cyp9M6* (1.19–2.13) and *Cyp9J32* (2.82–4.72) in Yaoundé samples. In Douala samples, overexpressed detoxification genes included *GSTD4* (1.34–55.3), *Cyp9J28* (2.23–7.03), *Cyp9M6* (1.49–2.59) and *Cyp9J32* (1.29–3.75). In Dschang populations, these seven detoxification genes were also found, but no overexpression was recorded.

## 4. Discussion

*Aedes aegypti* and *Aedes albopictus* are recognized as the main vectors of arboviruses in Cameroon [20,38,66,67]. Knowledge of their susceptibility and insecticide resistance profile is important for the implementation of successful vector control programs across the country. In the present study, the resistance profile to insecticides of both *Aedes aegypti* and *Aedes albopictus* from three urban settings in Cameroon was determined, and subsequently, genes and mechanisms conferring insecticide resistance in these vector populations were investigated. *Ae. albopictus* populations from the three sites were fully susceptible to permethrin and bendiocarb; however, a high resistance profile was detected against DDT in both Douala and Yaoundé. These results are in accordance with recent studies in the city of Douala and Yaoundé [39,58]. The rapid expansion of insecticide resistance in this species could result from domestic pollution or organic pollutants since *Aedes albopictus* is largely prevalent in water containers, spare tires, and discarded containers, which happen to be largely prevalent in agricultural cultivated sites [68]. It is also possible that this species susceptibility could have been affected by the increased use of insecticide repellents or through fumigation which is increasingly practiced in urban settings. Migration and founder effect could also be possible factors however these deserve further investigations [69,70].

*Aedes aegypti*, recorded alongside *Aedes albopictus,* was found much more resistant to pyrethroids in Douala. Similar observations were made previously [58]. The following supports high selection pressure by insecticides in *Aedes aegypti* compared to *Aedes albopictus*. It is likely that these species display different feeding and resting behavior, which could explain varying exposure to insecticides. Indeed, *Aedes aegypti* and *Ae. albopictus* have frequently been reported indoors. This particular behavior could have exposed them to the use of indoor base interventions such as insecticide sprays, aerosols, or treated nets. The high use of insecticides in households to prevent nuisance has been documented in different urban settings [71,72,73]. The high resistance profile in *Ae. aegypti* compared to *Ae. albopictus* has been reported in different epidemiological settings across West and Central Africa [17,74]. Very high resistance level to DDT was recorded in the different study sites even though this compound is no more used for vector control. It is possible that *Aedes* populations are still exposed to DDT through their use in agriculture or through long-term environmental persistence of organochlorine [73]. Adult mosquitoes in the three sites displayed high susceptibility to deltamethrin, particularly *Ae. Albopictus,* and could be explained by the fact that we used previous deltamethrin discriminating concentration of 0.05%, whereas the new tentative discriminating dose recommended for *Aedes* is now 0.03% [75].

Three kdr mutations F1534C, V1016G and V1016I out of the eleven previously detected in *Ae. aegypti* populations [53,76] were recorded in the present study. Some of these alleles (F1534C) were detected at an extremely high frequency close to fixation in Douala (90.0%) and at medium frequency (60.0%) in Dschang. The F1534C mutation was detected at a very high frequency (90%) compared to previous reports in the city of Douala (frequency 33.3%) [57] and suggests an increased expansion of this gene in Cameroon, which is the most represented across Africa [53]. Studies in Burkina Faso and Angola identified this allele close to fixation [53,77,78]; in Ghana, it was recorded with a frequency of 35.0% [79]. Two novel mutations V1016G and V1016I were also recorded. V1016I mutation was detected in Douala and Dschang. V1016G was also detected, albeit at a very low frequency in Douala (1.7%). The V1016I allele has previously been detected in Burkina Faso [77,78] and Ghana [80]. V1016G is largely distributed in Asia and V1016I in the Americas [53]. These genes could have emerged spontaneously or appeared through recent migration events in Africa [53]. The V1016G allele was reported to confer insensitivity to permethrin and deltamethrin, whereas the F1534C mutation was reported to confer resistance to permethrin [73,81]. The low resistance level to deltamethrin recorded during the present study could have resulted from the low frequency of V1016G allele in our samples and is consistent with previous findings elsewhere [73]. The three mutations S989P, V1016G and F1534C when occurring simultaneously in an individual were reported to confer a very high level of pyrethroid resistance [82,83]. It seems like resistance in *Aedes* mosquitoes is still not largely expanded across Central Africa, since *Ae. aegypti* mosquito populations from Congo [84] and Central African Republic [85] were reported to be free of these mutations.

*Aedes albopictus* mosquito population from Douala were found to carry the kdr F1354C mutation at a very low frequency (2.08%). This is the first detection of F1534C allele in *Ae. albopictus* mosquitoes in Cameroon and in Africa [57]. Thus far, three mutations at codon 1534 (F to C, L and S) have been reported in *Aedes albopictus*. The variant F1534S has been demonstrated to be moderately associated with resistance to DDT and pyrethroids [86,87]. As compared to *Aedes aegypti* which has eleven mutations occurring at 08 codons in the voltage-gated sodium channel, mutations on this gene in *Aedes albopictus* are less important since only four mutations affecting 02 codons (1532 and 1534) have been detected [53].

The expression levels of seven major detoxification genes (*Cyp6BB2*, *Cyp9J26*, *GSTD4*, *CCEae3a*, *Cyp9J28*, *Cyp9M6, Cyp9J32*) involved in insecticide resistance in *Ae. aegypti* were analyzed with recently developed multiplex TaqMan RT-qPCR assays. The P450s genes *CYP9J28*, *CYP9M6* and *CYP9J32* were significantly overexpressed in Yaoundé and Douala samples compared to the susceptible laboratory strain. CYP9J28 and CYP9J32 were recorded overexpressed in pyrethroid-resistant *Aedes aegypti* populations from Mexico, Peru and Cuba [53,88]. P450 detoxification genes including CYP9J10, CYP6BB2, CYP9J26 and CYP9J28 have been proven to metabolize pyrethroids [52,89] or to confer pyrethroid resistance when expressed transgenically in Drosophila [90]. From the review of Moyes et al. [53], it appeared that CYP6 and CYP9 genes were also the most commonly duplicated P450s genes in *Ae. aegypti,* suggesting that a copy number of variation may play an important role in differential expression phenotypes, although further studies are needed to confirm this hypothesis [53]. GSTD4 was found significantly overexpressed in the Douala *Aedes* population. GSTs alongside P450 genes have been reported to be involved in pyrethroid resistance in *Aedes aegypti* populations [19]. The present study is one of the few on *Ae. aegypti* in Africa, supporting at least partial involvement of metabolic base mechanisms in mosquito resistance to insecticides.

## 5. Conclusions

Target sites mutations and/or metabolic-based mechanisms were found to be associated with insecticide resistance in *Aedes aegypti* and *Aedes albopictus* in Cameroon. Although resistance is still not largely expanded in *Aedes* populations and does not affect all insecticide classes, the situation calls for immediate action in order to improve the control of *Aedes* populations. With the increasing number of arbovirus outbreaks in Cameroon and neighboring countries, it is becoming urgent that further strategies be implemented to improve vector control and prevent the spread of arboviral diseases.

## Figures and Tables

**Table 1:** Characteristics of the three study sites.

	Yaoundé	Douala	Dschang
Altitude above sea level	726 m	1 m	1500 m
Population size	2,765,568	2,768,436	301,385
Surface area	180 km^2^	210 km^2^	225 km^2^
Landscape	Congo Guinean equatorial forest	Coastal area	Highland area
Annual rainfall	1700 mm	4000 to 5000 mm	1364 mm

**Table 2:** Frequency of kdr resistance alleles in different populations of *Ae. aegypti* mosquitoes.

Population	Sample Size (Alleles)	Resistant Mutation Allelic Frequencies (Heterozygous/Homozygous Mosquitoes)			
		Pyrethroids/DDT			
		% F1534C	%V1016G	% V1016I	% S989P
Yaoundé	64	0.0 (0/0)	0.0 (0/0)	0.0 (0/0)	0.0 (0/0)
Douala	60	90.0 (6/24)	1.7 (1/0)	26.7 (14/1)	0.0 (0/0)
Dschang	20	60.0 (2/2)	0.0 (0/0)	60.0 (2/2)	0.0 (0/0)
Cameroon *Ae. aegypti* susceptible strain	40	0.0 (0/0)	0.0 (0/0)	0.0 (0/0)	0.0 (0/0)

*Aedes albopictus* mosquitoes were screened for kdr F1534C and the mutant allele was detected in Douala albeit at a very low frequency (2.08%) (Table 3).

**Table 3:** Incidence of resistance alleles in different populations of *Ae. albopictus* mosquitoes, assayed by TaqMan qPCR.

Population	Sample Size (Alleles)	Resistant Mutation Allelic Frequencies (Heterozygous/Homozygous Mosquitoes)
		Pyrethroids/DDT
		% F1534C
Yaoundé	48	0.0 (0/0)
Douala	48	2.08 (1/0)
Dschang	48	0.0 (0/0)
Cameroon *Ae. albopictus* susceptible strain	40	0.0 (0/0)

(a/b) = number of heterozygous/homozygous mosquitoes.

**Table 4:** Expression analysis of the detoxification genes analyzed in the three resistant *Aedes aegypti* mosquito populations compared to the susceptible mosquito strain.

Populations	Detoxification Gene Fold Changes (95% CI), *p* Value						
	*Cyp6BB2*	*Cyp9J26*	*GSTD4*	*CCEae3a*	*Cyp9J28*	*Cyp9M6*	*Cyp9J32*
Yaoundé	1.09 (0.773–1.48) *p* = 0.509	1.91 (0.936–3.88) *p*= 0.075	10.1 (0.630–39.0) *p* = 0.101	1.59 (0.961–2.61) *p* = 0.071	4.67 * (2.84–7.55) *p* < 0.001	1.56 * (1.19–2.13) *p* < 0.001	3.58 * (2.82–4.72) *p* = 0.032
Douala	0.556 (0.364–0.853) *p* < 0.001	1.621 (0.697–3.48) *p* = 0.298	9.34 * (1.34–55.3) *p* = 0.030	0.503 (0.251–1.01) *p* = 0.052	3.57 * (2.23–7.03) *p* < 0.001	1.88 * (1.49–2.59) *p* < 0.001	1.98 * (1.29–3.75) *p* < 0.001
Dschang	0.335 (0.122–0.890) *p* < 0.001	0.368 (0.235–0.649) *p* < 0.001	12.1 (0.840–40) *p* = 0.198	1.04 (0.900–1.183) *p* = 0.507	0.762 (0.251–2.27) *p* = 0.695	0.851 (0.655–1.14) *p* = 0.285	1.37 (0.518–3.74) *p* = 0.714

* indicate statistically significant overexpression (*p* < 0.05); 95% CIs are given in parentheses.

## Data Availability

All the data from the study is available in the manuscript.

## Supplementary Materials

The following are available online at https://www.mdpi.com/article/10.3390/genes12060828/s1, Figure S1: Agarose gel electrophoresis for total RNA extracted (presented for randomly selected samples); Table S1: Oligos used in the multiplex RT-qPCR assays for gene expression analysis and their analytical properties; Table S2: Primers, probes and control sequences used for TaqMan kdr genotyping
