# Supplementary material for: Analyses of Insecticide Resistance Genes in Aedes aegypti and Aedes albopictus Mosquito Populations from Cameroon
Source: Genes (Basel). 2021 May 28;12(6):828. doi: 10.3390/genes12060828 (PMC8229692; doi:10.3390/genes12060828)
Supplement: Supplementary file 1 [file genes-12-00828-s001.zip › genes-1165950-supplementary.pdf]

SUPPLEMENTARY INFORMATION

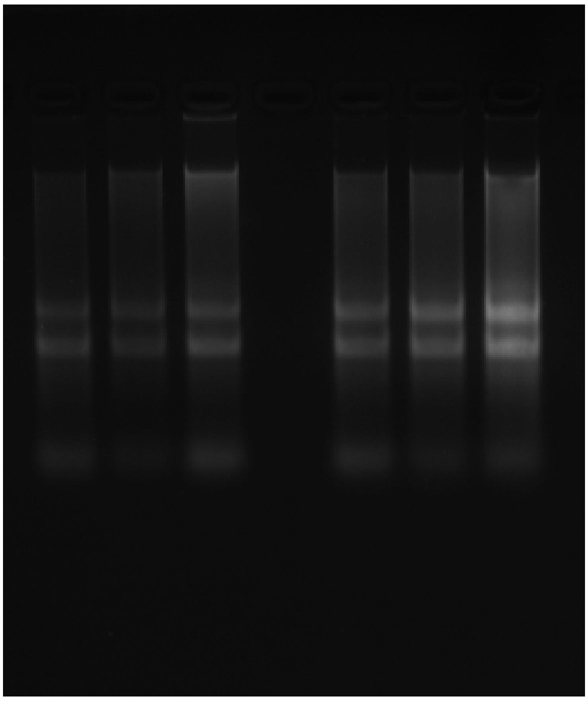

Suppl Figure S1 Agarose gel electrophoresis for total RNA extracted (presented for randomly selected samples). The presence of distinct ribosomal bands and the absence of degradation products shows that total RNA was intact, suitable for downstream analyses.

Supplementary Table S1 Oligos used in the multiplex RT-qPCR assays for gene expression analysis and their analytical properties

Multi-plex Detox Assay	Oligo	Sequence5′-3′	Dyes 5′-3′	Reaction conc. (nM)	%Eff	Ct Range (R^2)	%CV
(A)-(D) [norm.]	RPL8_F<br>RPL8_R<br>RPL8_P	GAAGGGAACCGTCAAGCAAATC<br>TCACGGAAGTGGACAACCG<br>ATGATCCAGGTCGTGGTGCCCCG	none<br>none<br>HEX-BHQ1	200<br>500<br>200	103.5-	24.8-34.2	3.00
(A)	CYP6BB2_F<br>CYP6BB2_R<br>CYP6BB2_P	GGCGAGGGAATCACGATGAA<br>GTACTTCCGTAGGGTTTCACTGAC<br>CCGTGAAGAAAATGAAACACTGCGCG	none<br>none<br>HEX-BHQ1	400<br>500<br>200	101.8	23.3-33.2	3.20
(A)	CYP9J26_F<br>CYP9J26_R<br>CYP9J26_P	ACAACAAATATCCTGGAGTGAAAGT<br>CGAGAACAGCGTCTTGCGAA<br>CGCGATCCGGAGTTGATCAAGC	none<br>none<br>ATTO647N-BHQ2	400<br>600<br>300	109.7	23.7-33.1	5.24
(B)	GSTD4_F<br>GSTD4_R<br>GSTD4_P	AGCCGGAATTTTTGAAGATCA<br>AGATGGCACGCGATTCG<br>ACGGTTCCCACACTGGCAGTAGGC	none<br>none<br>HEX-BHQ1	600<br>600<br>400	104.4	24.9-31.4	1.98
(B)	CCEae3a_F<br>CCEae3a_R<br>CCEae3a_P	TGGATGCAGTTTCCAAAACAC<br>GTGCACTCATGAGGGTTTCGTA<br>TAGGCTGTGTAGCAGA-GAGCGATGATGAAA	none<br>none<br>ATTO647N-BHQ2	500<br>600<br>500	100.0	26.3-36.4	0.69
(C)	CYP9J28_F<br>CYP9J28_R<br>CYP9J28_P	GACAAGTACCGAGGAGTCAAAGTTT<br>TTAACGGCCACCTGCTTGAT<br>ACGTACGTCATTCGCGATCCGGA	none<br>none<br>HEX-BHQ1	500<br>600<br>400	102.2	26.2-36.1	4.84
(C)	CYP9M6_F<br>CYP9M6_R<br>CYP9M6_P	CGTGATCTGTTTCAAAAGCTTGG<br>CCAACTGCTTTCCCCTTTTG<br>TCGGTGCACAATCCAAACAACGAGTT	none<br>none<br>ATTO647N-BHQ2	400<br>200<br>400	109.7	25.8-35.2	2.10
(D)	CYP9J32_F<br>CYP9J32_R<br>CYP9J32_P	CTACTTCCACGACAAGCCGATAC<br>GTCATATCAAACAG-TCCAAAAATCTTAGC<br>TTCCGCTCTTGGGCAGTACCGGTC	none<br>none<br>HEX-BHQ1	500<br>600<br>400	107.	26.2-35.6	6.77

F: Forward, R: Reverse, P: Probe; conc.: concentration: %Eff: % Efficiency (multiplex reaction) norm: normalizer. CV: Coefficient of Variation

Supplementary Table S2 Primers, probes and control sequences used for TaqMan kdr genotyping

Assay	Oligo Name	Sequence	Dye labels	Optimized Reaction concentration (nM)
kdr_F1534C	1534_F	CGAGACCAACATCTACATGTACCT	none	400
	1534_R	GTCGATGATGACACCGATGA	none	500
	F1534_P-wt	AACGACCCGAAGATGA	HEX - MGB	400
	1534C_P-mt	ACGACCCGCAGATGA	FAM - MGB	400
kdr_S989P	989_F	CATGATCGTGTTCCGGGTATT	none	300
	989_R	CACGTCACCCACAAGCATACA	none	600
	S989_P-wt	ATCGAATCCATGTGGGA	HEX - MGB	500
	989P_Probe-mt	AGTGGATCGAACCCA	FAM - MGB	50
kdr_V1016G/I	1016_Forward	ACCGACAAATTGTTTCCCAC	none	400
	1016_Reverse	GGACAAAAGCAAGGCTAAGAA	none	300
	V1016_P-wt	CACAGGTACTTAACCT	HEX - MGB	250
	1016G_P-mt1	CACAGGGACTTAACC	FAM -MGB	50
	1016I_P-mt2	CGCACAGATACTTAA	CY5 - MGB	600

F: Forward; R: Reverse; P: Probe; wt: wild type; mt: mutant

Notes:

1. For universal wild-type (FF, SS, VV) control use GB1 from the following list of sequences.
2. For mutant CC, PP, II control use GB2 from the following list of sequences.
3. For mutant GG control use GB3 from the following list of sequences.
4. For heterozygous FC, SP, VI control mix equal amounts of GB1 and GB2 from the following list of sequences.
5. For heterozygous VG control mix equal amounts of GB1 and GB3 from the following list of sequences.
6. For double mutant VI control mix equal amounts of GB2 and GB3 from the following list of sequences.

Name	Sequence	Type
**GB1**	TTCGCGAGACCAACATCTACATGTACCTCTACTTT-GTGTTCTTCATCATCTTCGGGTC<br>GTTCTTCAC-GCTGAATCTGTTCATCGGTGTCATCATCGACAATTCATGATCGTGTTCC<br>GGGTATTGTGCGGCGAGTGGATCGAATCCATGTGGGATTGTATGCTT-GTGGGTGAC<br>GTGTCCTACCGACAAATTGTTTCCCACTCGCACAGGTACTTAAC-CTTTTCTTAGCCTT<br>GCTTTTGTCC	Wild type swquence<br>-**F**1534<br>-**S**989<br>-**V**1016
**GB2**	TTCGCGAGACCAACATCTACATGTACCTCTACTTT-GTGTTCTTCATCATCTGCGGGTCG<br>TTCTTCAC-GCTGAATCTGTTCATCGGTGTCATCATCGACAATTCATGATCGTGTTCCGG<br>GTATTGTGCGGCGAGTGGATCGAACCCATGTGGGATTGTATGCTTGTGGGTGAC-GTGT<br>CCTACCGACAAATTGTTTCCCACTCGCACAGATACTTAACCTTTTCTTAGCCTTG<br>CTTTTGTCC	Mutant sequence1:<br>-1534**C**<br>- 989**P**<br>-1016**I**

**GB3**	TTCGCGAGACCAACATCTACATGTACCTCTACTTTGTGTTCTTCATCATCTTCG<br>GGTCGTTCTTCACGCTGAATCTGTTCATCGGTGTCATCATCGACAATTCATGA<br>TCGTGTTCCGGGTATTGTGCGGCGAGTGGATCGAATCCATGTGGGATTGTAT<br>GCTTGTGGGTGACGTGTCCTACCGACAAATTGTTTCCCACTCGCACAGGGA<br>CTTAACCTTTTCTTAGCCTTGCTTTTGTCC	Mutant sequence2:<br>1016**G**<br>(Wild type for the rest)
